# Endoscopic Negative Pressure Therapy (ENPT) Is Superior to Stent Therapy for Staple Line Leak After Sleeve Gastrectomy: a Single-Center Cohort Study

**DOI:** 10.1007/s11695-021-05287-z

**Published:** 2021-03-01

**Authors:** Rami Archid, Fateh Bazerbachi, Barham K. Abu Dayyeh, Felix Hönes, Suhaib J. S. Ahmad, Karolin Thiel, Giorgi Nadiradze, Alfred Königsrainer, Dörte Wichmann

**Affiliations:** 1 Department for General, Visceral and Transplant Surgery, Eberhard-Karls-University Hospital, Hoppe-Seyler-Str. 3-5, 72076 Tuebingen, Germany; 2 Division of Gastroenterology, Department of Medicine, Massachusetts General Hospital and Harvard Medical School, Boston, MA 02114 USA; 3 Department of Gastroenterology and Hepatology, Mayo Clinic, Rochester, MN 55905 USA; 4 Milton Keynes University Hospital, Milton Keynes, UK

**Keywords:** Sleeve gastrectomy, Surgical complications, Staple line leak, Endoscopic negative pressure therapy, Endoscopic Vacuum therapy, Stent

## Abstract

**Purpose:**

Staple line leak (SLL) is a serious complication after sleeve gastrectomy (SG). Common endoscopic treatment options include self-expandable metallic stent (SEMS), endoscopic internal drainage (EID), and endoscopic closure. The endoscopic negative pressure therapy (ENPT) is a promising treatment option combining temporary sealing of the defect with drainage of the inflammatory bed. In this study, we compare the outcome of ENPT and SEMS for the treatment of SLL following SG.

**Materials and Methods:**

A retrospective cohort of 27 patients (21 females) treated at a single center for SLL after SG was included. ENPT was primary therapy for 14 patients and compared with 13 patients treated primarily using SEMS.

**Results:**

ENPT was associated with a significant reduction of hospital stay (19 ± 15.1 vs. 56.69 ± 47.21 days, *p* = 0.027), reduced duration of endoscopic treatment (9.8 ± 8.6 vs. 44.92 ± 60.98 days, *p* = 0.009), and shorter transabdominal drain dwell time (15 (5–96) vs. 45 (12–162) days, *p* = 0.014) when compared to SEMS. Whereas endoscopic management was successful in 12/14 (85.7%) of patients from the ENPT group, SEMS was successful in only 5/13 (38.5%) of patients (*p* = 0.015). Furthermore, ENPT was associated with a significant reduction of endoscopic adverse events compared with SEMS (14.3% vs. 76.92% *p* = 0.001).

**Conclusion:**

Compared with SEMS, ENPT is effective and safe in treating SLL after SG providing higher success rates, shorter treatment duration, and lower adverse events rates.

## Introduction

Bariatric surgery is the most effective option for individuals suffering from obesity and related diseases [1]. Among the most popular bariatric procedures is the laparoscopic sleeve gastrectomy (SG) [2]. SG accounted for 58% of all bariatric procedures performed in the USA in 2016, representing a 346% rise compared with 2011 [3].

Nevertheless, as with any other surgical procedures, SGs present various possible complications, with the most serious being the staple line leak (SLL). Despite a low prevalence of 1.5%, SLL is associated with high morbidity and mortality and is difficult to prevent, predict, and manage [4, 5].

SLL-associated infections and discontinuity of the stomach wall can lead to sepsis, chronic fistula formation, and mortality [6]. SLL is among the most common cause of death after SG, with leak-related mortality of 0.1% and an overall mortality rate after SG of 0.3% [7]. Thus, early diagnosis and rapid management are paramount.

To date, guidelines regarding the optimal management of staple line leaks are lacking. Given that surgical closure is not effective, therapeutic paradigms shift towards endoscopic interventions [4, 7], with salvage gastrectomy as a rescue measure [8]. Endoscopic options, such as self-expandable metallic stent (SEMS), application of over-the-scope-clip (OTSC**®**), and endoscopic intraluminal suturing of leaks are based on the mechanical closure or bridging of the SLL, often after a percutaneous or surgical drainage [9]. SEMS are among the most popular treatment strategies [6, 10, 11].

However, interest is growing in endoscopic treatments of leaks using internal drainage of the collection, without closure of the defect [9]. Endoscopic internal drainage (EID), e.g., using double-pigtail plastic stents (DPS), is associated with success rates of nearly 75% in large cohorts [9].

The endoscopic negative pressure therapy (ENPT) combines the temporary sealing of the defect with drainage of the inflammatory bed. ENPT has been successfully used for the treatment of various types of gastrointestinal injuries and anastomotic leaks, e.g., after esophagectomy [12, 13], after rectal resection [14, 15], in the duodenum or pancreas [16]. ENPT is also a viable option for patients with SLL owing to its tolerability and effectiveness [5, 16].

Although ENPT has demonstrated superiority compared with SEMS in the management of upper GI transmural defects [17], to the best of our knowledge, data is lacking regarding this performance following bariatric surgery staple line and anastomotic leaks. We aim to compare the outcomes of ENPT and SEMS for the treatment of SLL following SG.

## Materials and Methods

### Study Design

All procedures performed in studies involving human participants were in accordance with the ethical standards of the institutional and/or national research committee and with the 1964 Helsinki declaration and its later amendments or comparable ethical standards. This study was approved by the ethics committee of the University Hospital of Tuebingen, Germany (380/2018BO2); informed consent does not apply. A cohort study design was conducted to compare the outcomes of ENPT and SEMS for the treatment of SLL following SG. We included all SLL following SG, which were managed endoscopically in our institution between January 2009 and June 2020. We excluded cases managed non-endoscopically and cases treated at outside hospitals and later transferred to our institution. SGs performed outside our hospital were included in the study if they were treated with SLL in our hospital.

Data were analyzed from a prospectively maintained endoscopic database, including morphology, occurrence time, size and location of leaks, existence and size of the intra-abdominal collection, type of intervention, length of intensive care unit (ICU) stay, length of hospital stay, and time to recovery, and were collected. Sequential Organ Failure Assessment (SOFA) [18] score was assessed in ICU patients and the Charlson comorbidity index score [19] for all patients upon symptomatic presentation. Patients were followed for ≥ 3 months after discharge.

Until September 2014, SEMS were used for the treatment of SLL after SG. In October 2014, we shifted our therapeutic strategy towards ENPT. Both treatment groups were evaluated and compared. Internal drainage via stenting was not implemented in our institution.

The primary outcome was the success of endoscopic treatment, defined as the rate of leak resolution as determined by endoscopy, cross-sectional imaging, and clinical course. Secondary outcomes were complication rates and duration of therapy.

### Sleeve Gastrectomy

The sleeve gastrectomy technique has been previously described [5]. A 42-Fr bougie was positioned along the lesser gastric curvature. Until November 2013, a 34-Fr bougie was used. The gastric sleeve was performed strictly along the bougie using a 60-mm stapler (Ethicon Endo-Surgery Inc., Cincinnati, OH) and until November 2019 with a bio-absorbable staple line reinforcement. SGs performed outside hospitals lacked procedural details.

### Treatment Strategy

**Fig. 1:**
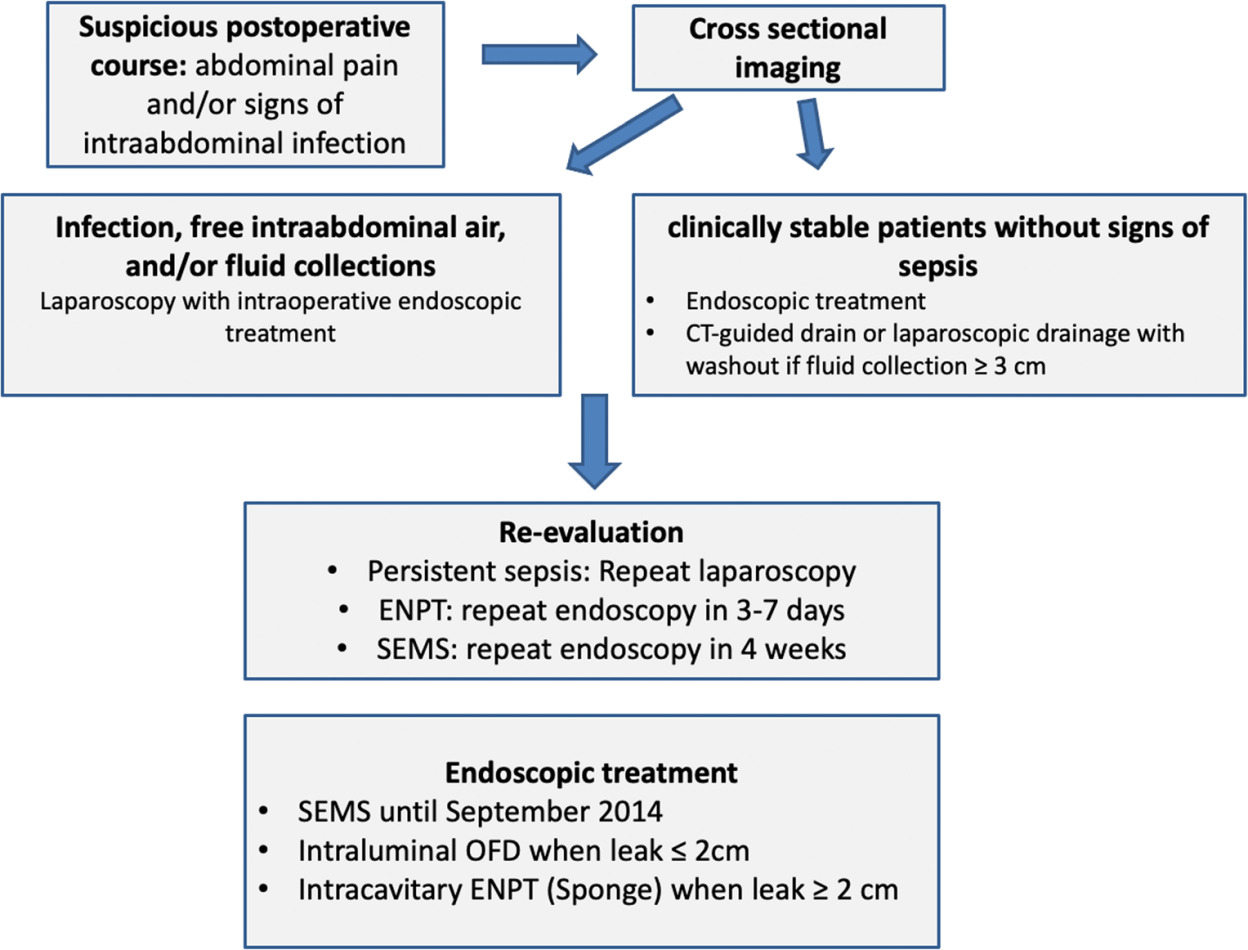
Flowchart depicting therapeutic strategy for SLL evaluation and management

Our therapeutic paradigm is depicted in Fig. 1. All patients presenting with upper abdominal pain and signs of infection or sepsis after SG undergo an urgent CT scan.

Additionally, in clinically stable patients without signs of sepsis, a CT-guided drain or laparoscopic drainage is performed only in collections or abscesses larger than 3 cm. However, in patients presenting with severe sepsis, a diagnostic laparoscopy with drainage is performed to provide abdominal washout.

Whenever possible, index endoscopy is performed during laparoscopy, allowing for optimal leak visualization and early index endoscopic treatment.

### Endoscopic Procedures

All endoscopic procedures were performed under monitored anesthesia care or general endotracheal intubation. Endoscopy was performed using a standard gastroscope with an outer diameter of 9.7 mm.

### Endoscopic Negative Pressure Therapy

**Fig. 2:**
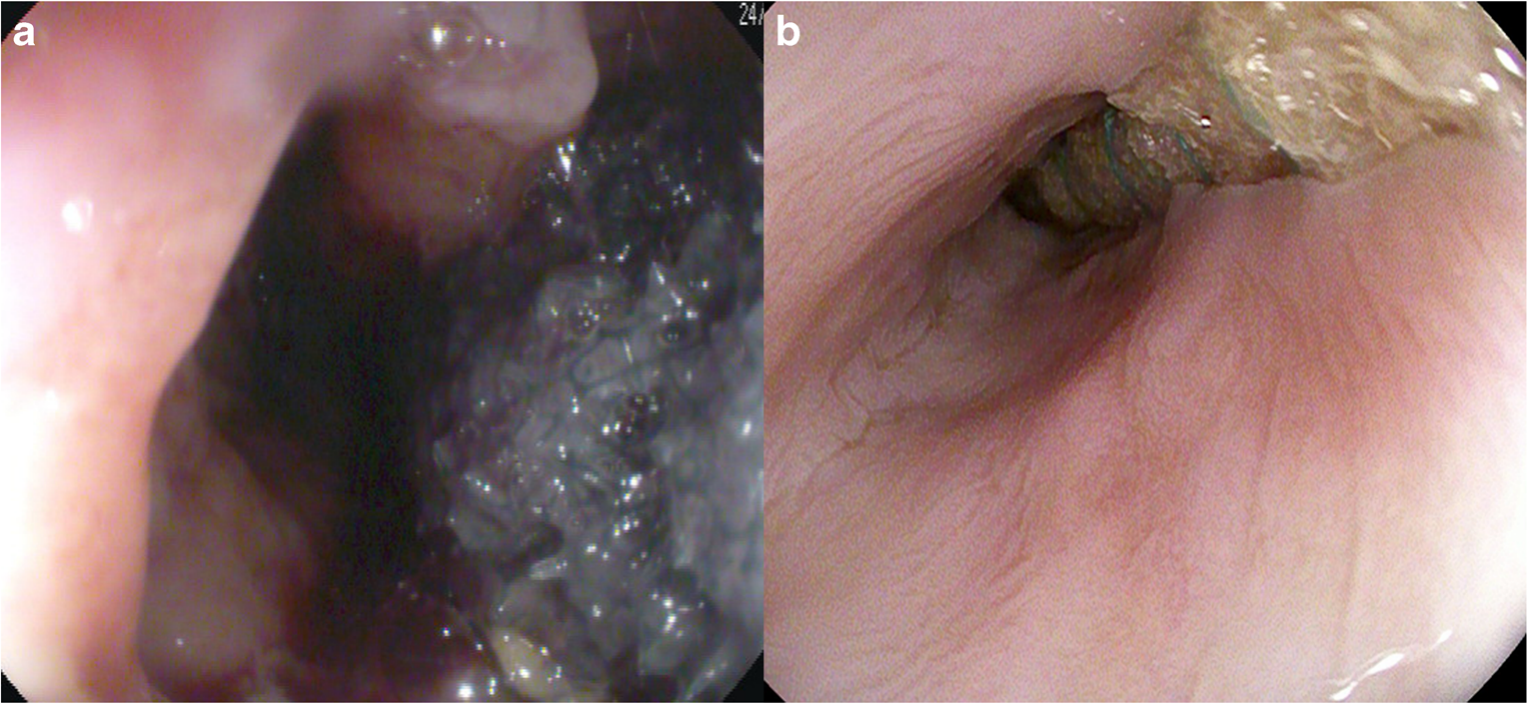
Endoscopic imaging during insertion of intracavitary ENPT using open-pore polyurethane foam drainage (OPD) into the leak cavity (**a**) and of intraluminal ENPT using open-pore film drainage (OFD) lying within the lumen next to the staple line leak (**b**)

As described previously [5], the commercially available open-pore polyurethane foam drainage (OPD) system Eso-SPONGE® (B. Braun Melsungen AG, Melsungen, Germany) was used for *intracavitary* ENPT (Fig. 2a). For *intraluminal* therapy, as previously described in video detail [20, 21], an open-pore film drainage (OFD) was fashioned by wrapping and suturing a very thin open-pore double-layered drainage film (Suprasorb CNP, Drainage Film; Lohmann & Rauscher International GmbH & Co.KG, Rengsdorf, Germany) on the gastric segment of a nasojejunal feeding tube (Freka® Trelumina, Fresenius Kabi Deutschland GmbH, Bad Homburg Germany) (Fig. 2b). The OFD device was placed intraluminally in the gastric sleeve and assembled to cover the leak area overlapping the healthy staple line by at least 2 cm. The distal segment of the tube was used for enteral feeding. The OFD was placed under endoscopic or fluoroscopic guidance over a guidewire. A continuous Vacuum of − 125 mmHg was generated by an electronic device (KCI V.A.C. Freedom; KCI USA Inc., San Antonio, Texas, USA). Endoscopy was repeated after 3–7 days. In the case of a persisting leak or in the case of uncertainty, an ENPT system was re-inserted, and the treatment was continued.

### Self-expandable Metallic Stent

SEMS was placed over a stiff flexible guidewire that was introduced endoscopically to the small intestines. Fully covered (FC) SEMS (12.5 cm in length, 23 to 28 mm in diameter) was used in all stented patients, except six patients treated between 2013 and 2014 with stents measuring 23 cm in length and 24/28 mm in diameter, specially designed for stenting in bariatric surgical sleeve gastrectomy.

The stents were positioned starting 5 to 10 cm proximal from the detected leak onwards into the stomach if an esophageal stent was used or into the duodenal bulb if the bariatric designed stent was used, and the stent was fixated with hemoclips at the proximal margin.

### Intrapyloric Botulinum Toxin (Botox) Injection

In five cases, four in the ENPT group, and one in the SEMS group, an injection of 100 units of botulinum toxin diluted in 10 cc of saline into the pylorus was performed during endoscopy, aiming to reduce the intragastric pressure [22].

### Statistical Analysis

All analyses were performed using SPSS v. 24.0.0.1 (IBM, Armonk, NY, USA). Data were tested for normality and presented as means ± SD, (range), or median (range) or percentages for categorical data. The Mann-Whitney *U* test test was performed for comparing means and chi-square test for comparing frequencies between the study groups. All reported *p* values were 2 tailed, and *p* values of less than 0.05 were considered statistically significant. An intention-to-treat analysis was pursued for both groups.

### Case Series Risk of Bias

We relied on a newly published tool to provide a quality assessment of the risk of bias in our reported case series [23]. This tool has been widely used and applied in previous publications, with consistency among reviewers [24–28]. All patients represented the whole experience of our center during the study period and case inclusion was not biased (all qualified patients were included without omission). The exposure (ENPT/SEMS) was ascertained for all cases. The outcome (defect healing) was adequately ascertained in all cases. No alternative causes explained the outcome of healing. Follow-up was adequate for the assessment of the outcome.

## Results

### Baseline Characteristics

A total of 27 patients (21 females, 6 males) were treated for SLL after SG (13 with SEMS until October 2014 and 14 with ENPT thereafter) between January 2009 and June 2020 and included in the final analysis for this study. SEMS was used as primary treatment in 13 patients. In 1 ENPT failure, the patient was treated with SEMS; however, laparoscopic gastrectomy was eventually needed.

Both groups were similar in baseline characteristics, as shown in Table 1.

**Table 1:** Patients characteristics

	ENPT	SEMS	*P*
Sex (female:male)	10 (71.4%):4 (28.6%)	9 (69.2%):4 (30.8%)	0.90
Age	44.98 ± 16,74	42.01 ± 11.47	0.59
BMI	51.51 ± 10.57	49.98 ± 9.97	0.71
Comorbidities (*n*, percentage)			
DMII	5 (35.71%)	3 (23%)	0.47
Hypertension	4 (28.57%)	4 (30.7%)	0.90
GERD	3 (21.42%)	4 (30.7%)	0.58
Eating disorder	4 (28.57%)	3 (23%)	0.74
Smoking	1 (7.14%)	0 (0%)	0.32
Lung fibrosis	1 (7.14%)	0 (0%)	0.32
Charlson comorbidity index	1.07 ± 1.27, (0–4)*	1 ±1.08, (0–3)	0.97
No bariatric or gastric procedures prior to SG	14/14 (100%)	11/13 (84.6%)	0.127
SG performed at our institution	12/14 (85.7%)	8/13 (61.5%)	0.152

*Mean ± SD, (range)

### Leak Characteristics

Leaks were diagnosed during index admission in only two patients from each group. The majority of patients in both study groups presented with late leaks (> 8 days after SG). Patients from ENPT and SEMS group presented symptomatically 13 (0–61) (IQR 30) days and 11.50 (0–296) (IQR 19) days after surgery, respectively. The majority of leaks were located in the upper part of the gastric sleeve and were < 5 mm. As demonstrated in Table 2, there were no significant differences between both groups regarding the clinical data and the main characteristics of the leaks.

**Table 2:** Clinical characteristics

	ENPT	SEMS	P
Time interval between SG procedure and diagnosis of leak (days)	25.50 ± 22.67, (3–61)	45 ± 83.83, (1–296)	0.73
Diagnosis of leak during index admission for SG	2/14 (14.3%)	2/13 (15.4%)	0.56
Detection of leak in CT scan	9/14 (64.3%)	9/13 (69.2%)	0.34
Detection of leak in endoscopy	5/14 (35.7%)	4/13 (30.8%)	0.78
Pathological CT scan findings			
Not documented	0	1 (7.7%)	0.542
Not suspicious for leak	1 (7.1%)	0	
Collection/abcess < 2cm	2 (14.3%)	0	
Collection/abcess 2–5cm	3 (21.4%)	2 (15.4%)	
Collection/abcess > 5cm	2 (14.3%)	4 (30.8%)	
Extraluminal air	4 (28.6%)	5 (41.7%)	
Extraluminal oral contrast	1 (7.7%)	1 (7.7%)	
Detection of leak during index endoscopy	14/14 (100%)	13/13 (100%)	--
Location of leak			
G-E junction	11 (78.6%)	13 (100%)	0.20
G-E junction and mid gastric	2 (14.3%)	0	
Distal gastric	1 (12.5%)	0	
Leak onset [29]			
Early (d 1–3)	1 (7.1%)	1 (7.7%)	0.83
Intermediate (d 4–7)	2 (14.3%)	3 (23.1%)	
Late (d 8 or more)	11 (78.6%)	9 (69.2%)	
Number of leaks			
One	12 (85.7%)	11 (84.6%)	0.99
Two	1 (7.1%)	1 (7.7%)	
Three	1 (7.1%)	1 (7.7%)	
Size of leak			
< 5 mm	9 (64.3%)	7 (63.6%)	0.64
5–10 mm	5 (35.7%)	4 (36.4%)	
Concomitant sleeve stenosis			
Yes	1 (7.1%)	3 (23.1%)	0.26
No	13 (92.9%)	10 (76.9%)	

### Therapy Data and Outcomes (Table 3)

Endoscopic was initiated immediately in most patients after detecting SLL in both groups, median 0 days (0–18 days).

**Table 3:** Therapy data

	ENPT	SEMS	*P*
Time interval between diagnosis of a leak and endoscopic treatment (days)	0.7 ± 0.27, (0–1)	2 ± 5.24, (0–18)	0.19
Time interval between bariatric operation and endoscopic treatment (days)	25.64 ± 22.79, (3–62)	48.17 ± 87.22, (5–296)	1.0
Initial treatment strategy			
Only endoscopic treatment (ENPT or SEMS)	10 (71.4%)	12 (92.3%)	0.00
(ENPT or SEMS) with laparoscopic drainage	3 (21.4%)	1 (7.7%)	
(ENPT or SEMS) with CT drainage	1 (7.1%)		
Endoscopic treatments			
ENPT alone	13/14 (92.9%)	--	
ENPT followed by SEMS	1/14 (7.1%)	--	
SEMS alone	--	7/13 (53.8%)	
SEMS followed by OTSC	--	6/13 (46.2%)	
Number of treatments needed	2 (1–10) ENPT	2 (1–3) SEMS	0.57
Duration of endoscopic treatment (for ENPT or SEMS) (days)	7.29 ± 7.43, (1–28)	44.92 ± 60.98, (7–234*)	0.0004
Number of OTSC	0	0 (0–3)	0.012
Number of endoscopies needed pro patient	3.79 ± 3.12, (1–11)	6.23 ± 4.41, (2–16)	0.012
Laparoscopy performed during treatment in	6/14	12/13	0.006
Numbers of laparoscopies needed per patient	0.93 ± 1.27, (0–3)	1.46 ± 0.88, (0–3)	0.10
ICU surveillance	7/14 (50%)	9/13 (69.2%)	0.31
SOFA Score in ICU patients	3.71 ± 3.3, (0–6)	3.33 ± 1.8, (0–6)	0.67
Duration of stay at ICU (days)	4.86 ± 9.32, (0–30) (IQR7)	15.08 ± 29.78, (0–107) (IQR13)	0.09
Duration of stay at hospital (days)	22.71 ± 24.48, (5–96)	56.69 ± 47.21, (12–162)	0.014
Duration of transabdominal drain (days)	27.86 ± 46.60, (5–96)	96.10 ± 76.17, (12–162)	0.009
Intrapyloric Botox injection performed in	4/14 (28.5%)	1/13 (7.7%)	0.16
Pneumatic dilatation of pylorus performed in	1/14 (7.1%)	0/13 (0%)	0.32
Complications of endoscopic treatment	2/14 (14.3%)	10/13 (76.9%)	0.001
Bleeding	1/14 (7.1%)	1/13 (7.7%)	0.95
Dislocation	0	1 (7.7%)	
Ingrowth	0	1 (7.7%)	
Fistula	0	0	
Death	1/14 (7.1%)		0.32
Outcome and change of treatment strategy			
Successful endoscopic treatment	12/14 (85.7%)	5/13 (38.5%)	0.015
Surgical suturing	--	1/13 (7.7%)	
Gastrectomy	1/14 (7.1%)	7/13 (53.8%)	
Death	1/14 (7.1%)	0	

*An uncovered stent was placed and overstented twice before referral to our department, where gastrectomy was performed after 234 days of stent treatment. Risk of bias: our case series showed a low risk of bias in totality

ENPT application was associated with a significant reduction in-hospital stay (*p* = 0.014), as well as a reduced duration of endoscopic treatment (*p* = 0.0004), the need for fewer endoscopies (*p* = 0.012), and shorter percutaneous drainage placement period (*p* = 0.009) when compared to the SEMS group. Additionally, there was a trend in the ENPT group towards fewer laparoscopies (*p* = 0.10) and shorter ICU stay compared with SEMS.

Whereas endoscopic management was successful in 12/14 (85.7%) of patients from the ENPT group, SEMS was successful in only 5/13 (38.5%) of patients (*p* = 0.015). Therefore, surgery was required in only one patient (7.1%) from the ENPT group and in 8 (61.5%) patients from the SEMS group (53.8% gastrectomy and 7.7% surgical suturing).

Although ENPT was associated with a significant reduction of endoscopic treatment-associated complications (Table 3), when compared to SEMS (12.5% vs. 76.92% *p* = 0.027), death occurred in one patient from the ENPT group, whereas all 13 patients from the SEMS survived. However, the difference in mortality was not significant between study groups (*p* = 0.32). The patient who died suffered from severe comorbidities related to dilated cardiomyopathy, idiopathic lung fibrosis, and pulmonary hypertension, and perished from uncontrolled sepsis.

## Discussion

There is currently no standard algorithm for the endoscopic management of SLL. Therefore, comparative studies of different treatment strategies are relevant to understand the management implications of this serious complication.

There is an increasing trend for a change in clinical practice from a strategy of diversion with SEMS towards an open endoscopic treatment of leaks using active or passive internal drainage of the collection with aggressive management of distal sleeve stenosis and without closure of the defect [9]. In this study, we show a higher success rate, shorter treatment duration, and a lower complication rate when using ENPT compared with SEMS when treating SLL.

Indeed, SEMS has been associated with delayed cure (median of 310 days), higher complications, and failure rates up to 84.6% in SLL management [6, 30]. Moreover, prolonged treatment duration, ICU stay, and the need for repeated therapeutic and diagnostic interventions make endoscopic treatment strategies using SEMS, DPS, and OTSC demanding and expensive with costs of nearly 75,000 Euro per patient in a recent French series [31].

In general, ischemia and inflammation of tissue surrounding leaks are responsible for the poor integrity of the tissue and result in high failure rates of mechanical closure devices [9]. However, high success rates in chronic leaks and fistulas have been reported for sleeve defect closure using a cardiac septal defect occluder, which promotes tissue growth while sealing the fistula tract [32].

Given that the high-pressure milieu within the gastric sleeve is a major factor for the development and recalcitrance of staple line leak [8], different treatment strategies such as performing a fistula-jejunostomy [33], a salvage total gastrectomy with oesophago-jejunostomy, and placing an endoscopic internal drainage (EID) help to overcome the high-pressure system with beneficial effects on the course of SLL. However, surgery might be challenging and is associated with several complications when performed in an infected field.

ENPT facilitates the healing process of the staple line and anastomotic leaks similarly to the healing of open wounds under vacuum therapy. Important factors contributing to the beneficial effects of this treatment modality are constant suction of wound secretion and debris along with the collapse of the lumen, transportation of infectious material, resolution of interstitial edema, modification of signaling milieu, simulation of tissue perfusion and wound granulation, rapid fibrin deposition, and epithelialization resulting in secondary wound closure of the defect, while the secretions are constantly suctioned through the drainage tube. This constant mobilization of fluids may help control intra-abdominal and general sepsis, endoscopic restoration of staple line continuity [16, 20].

Stent therapy can be complicated by stent occlusion, migration, tissue overgrowth, and poor tolerability [9]. In the ENPT group, bleeding occurred only in one patient, from the short gastric vessels 1 day after initiating intracavitary ENPT using OPD. This patient was treated with angiographic embolization, and intracavitary ENPT was changed to intraluminal open-pore polyurethane foam drainage (OFD). One patient in the ENPT group perished on day 23, this patient suffered from lung fibrosis, severe pulmonary hypertension, and dilated cardiomyopathy. Mortality was attributed to worsening cardiomyopathy secondary to severe sepsis. Considering the median time of 5 days for resolution of leaks in the ENPT group, it is likely that the healing conditions were dramatically worsened due to the cardiac and pulmonary comorbidities, so that healing was not achieved within 18 days of ENPT. It is unlikely that mortality, in this case, would have been prevented with treatment within the SEMS arm.

The relatively large diameter (15–30 mm) of the foam-based OPD may hinder endoscopic placement through small defect openings. Therefore, the newly developed OFD, with a 4- to 6-mm diameter, is preferred for placement either through small defects and for intraluminal placement. Moreover, OFD is thought to offer better adhesiveness characteristics, easy removal, and less damage to the surrounding tissue [5, 20]. In addition, intraluminal ENPT using OFD provides effective and reliable mobilization of intraluminal bile, which has a deleterious effect on wound healing [20].

Success rates of up to 93% for EID using DPS are reported in a recent study combining EID with laparoscopic external drainage. However, long-term treatment (median 120 days) and the need for repeated endoscopic interventions are a limiting factor [34]. Moreover, the passive drainage from the inflammatory side using EID towards the lumen might not work when leaks are located in the thorax due to the inspiration-associated negative pressure towards the extraluminal cavity. EID is not recommended in the disorganized or uncontained collection [9]. Complications, such as ulcerations, dysphagia, discomfort, and splenic hematoma, have been reported with the use of EID [9, 35]. Another important therapeutic modality is endoscopic septotomy, which entails the incision and enlargement of the leak opening to allow passive internal drainage into the gastric lumen, and has been successful in refractory leaks [36].

Our study has major limitations highlighted by the retrospective nature and limited numbers of patients and a possible era effect in the implementation of the two modalities. However, study groups were similar in baseline characteristics and leak manifestations. There were no significant differences between study groups in Charlson comorbidity index score, proportion of ICU patients after symptoms onset, or in Sequential Organ Failure Assessment (SOFA) score for ICU patients. Nonetheless, existing differences between the study groups are potential confounders as in the case of presence of collection cavity > 5 cm (30.8% vs. 14.3%) as well as the concomitant presence of distal sleeve stenosis (23.1% vs. 7.1%) for the SEMS and ENPT group respectively, although not statistically significant. Given these limitations, and although promising in results, the comparison between ENPT and SEMS should be considered exploratory, awaiting data from larger studies.

An inter-group change between the two endoscopic treatment strategies occurred only in one patient, who received SEMS treatment after ENPT and finally underwent gastrectomy because of failed endoscopic management after 28 days of ENPT and 30 days of treatment using SEMS. Furthermore, the change from 34-Fr tube to 42-Fr tube for calibration of gastric sleeve since November 2013 and the use of botulinum toxin in sporadic cases should be considered as potential confounders of study results, in addition to other unknown confounders precipitated by an era effect. Treatment success in the SEMS group of this study was lower than in a published meta-analysis, where the success rate of 72.8% (24 studies) was reported [37]. Leaks in the SEMS group in our study were delayed in the majority of cases. It is possible that this chronological difference in leak characteristics between our study and others could be partially responsible for the lower effectiveness of this treatment strategy in our cohort. Furthermore, mean stent sojourn time was higher in the meta-analysis compared with our study. Higher success rates cannot be excluded after a more extended treatment period. However, higher complication rates and lower tolerance of stents should also be considered.

In conclusion, an endoscopy is a powerful tool in managing complications after bariatric surgery, and ENPT may be a promising approach to manage selected cases of the feared sleeve gastrectomy leaks. This therapy may be preferred to SEMS as a first step in managing these dreaded complications, with the versatility of being incorporated as part of hybrid therapy, such as ENPT with simultaneous stenting (https://clinicaltrials.gov/ct2/show/NCT03962179). These endoscopic therapies may become pivotal in the management of bariatric surgical complications in a multidisciplinary program involving surgeons, gastroenterologists, and interventional radiologists.

## Abbreviations

DPS: Double-pigtail plastic stents
EID: Endoscopic internal drainage
ENPT: Endoscopic negative pressure therapy
OFD: Open-pore film drainage
OPD: Open-pore polyurethane foam drainage
OTSC**®**: Over-the-scope-clip
SEMS: Self-expandable metallic stent
SG: Sleeve gastrectomy
SLL: Staple line leak
